# Startling responses of zebrafish: an interview with Harold Burgess

**DOI:** 10.1186/s12915-018-0589-1

**Published:** 2018-11-01

**Authors:** Harold A. Burgess

**Affiliations:** Division of Developmental Biology, Eunice Kennedy Shriver National Institute of Child Health and Human Development, Bethesda, MD 20892 USA

**Keywords:** Neurobiology, Behavior, Zebrafish, Neural circuits, Neurodevelopment

## Abstract

Harold Burgess is a Senior Investigator at the *Eunice Kennedy Shriver* National Institute of Child Health and Human Development (NICHD), part of the National Institutes of Health. Work in his lab combines genetic and imaging techniques to study neural circuits required for sensory guided behavior in zebrafish. In this interview Harold shares his thoughts on the changing field of neural development, pre-publication review, and ‘Darwinian experiments’ of peer review.

## What are your current research interests?

My lab decodes neural circuits involved in sensorimotor processing, and how the functional development of these circuits is altered by disease-related gene mutations. Specifically, we study the neuronal pathways that link sudden threatening stimuli to startle responses, and how transmission along these pathways is influenced by environmental conditions and behavioral state. We use larval-stage zebrafish for these studies because their small size and transparency lets us image the entire brain using a standard confocal microscope—that way we can systematically analyze brain structure and function rather than just characterizing candidate neurons or areas.

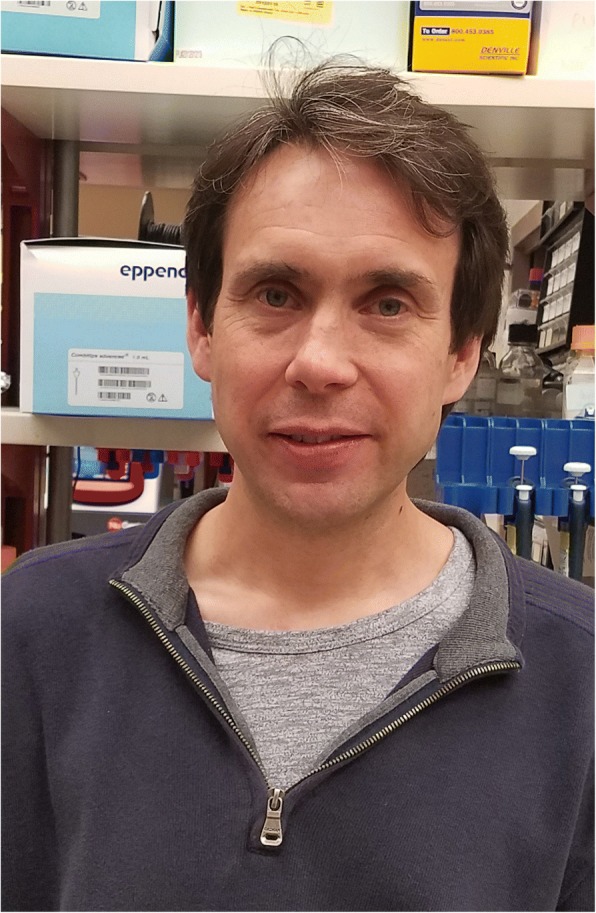



## What are your predictions for the field over the next 5 years?

Around 15–20 years ago, many labs that were leaders in studying neural development started applying their molecular genetic expertise to the functional analysis of neural circuits for behavior. In part, I think this was motivated by the feeling that many of the key molecular players in neural development had been identified and the excitement of moving into a fresh field. Over the last few years, large patient cohorts and next-gen sequencing approaches have started to provide really solid leads into genetic changes linked to neurodevelopmental disorders—and, perhaps no surprise, many of them are old friends from the neural development field. I think that the opportunity to link genotype to phenotype at the level of neuronal pathways closely linked to behavior will inspire a resurgence of interest in studying the functional development of neural circuits.

## What motivates you to provide peer review for journals?

I generally review papers simply out of a sense of fairness. I multiply the number of papers I anticipate submitting in a given year by three, and try to ensure I review at least that number of manuscripts each year! Occasionally I’m also invited to review a paper with an abstract too cool to pass up—those papers usually move to the front of my to-do list and get a blazingly fast review.

## What changes, if any, would you make to the current system of peer review?

We seem to be in the middle of a great Darwinian experiment in peer review—there has been tremendous diversification in the publishing world and there’s now a large ecosystem of journals with very different approaches to review. As labs submit and review papers under different paradigms, perhaps a consensus as to an optimal system or systems may emerge. I’m a fan of the opportunity to post research pre-publication, for example, on the bioRXiv server. We’ve received really useful feedback that has helped strengthen the submitted paper.

## Have you had any memorably good or bad experiences of peer review, as an author or as a reviewer?

Most of my experiences as an author have not cultivated a love for humanity. To satisfy reviewer number 3 I’d have to board up the lab and hide under a rock in the arctic tundra. My best experience was when our studies unexpectedly led to a study of muscle development. The courteous criticisms by the reviewers of that paper were so appreciated I was briefly tempted to switch fields!

### Website:

http://ubn.nichd.nih.gov/.

